# Phenotyping pipeline reveals major seedling root growth QTL in hexaploid wheat

**DOI:** 10.1093/jxb/erv006

**Published:** 2015-03-04

**Authors:** Jonathan A. Atkinson, Luzie U. Wingen, Marcus Griffiths, Michael P. Pound, Oorbessy Gaju, M. John Foulkes, Jacques Le Gouis, Simon Griffiths, Malcolm J. Bennett, Julie King, Darren M. Wells

**Affiliations:** ^1^Centre for Plant Integrative Biology, School of Biosciences, University of Nottingham, Sutton Bonington LE12 5RD, UK; ^2^Department of Crop Genetics, John Innes Centre, Norwich Research Park, Norwich NR4 7UH, UK; ^3^Division of Plant and Crop Sciences, School of Biosciences, University of Nottingham, Sutton BoningtonLE12 5RD, UK; ^4^INRA, UMR 1095 Génétique, Diversité et Ecophysiologie des Céréales, 63100 Clermont-Ferrand, France

**Keywords:** High-throughput phenotyping, root system architecture.

## Abstract

Seedling root traits of wheat (*Triticum aestivum* L.) have been shown to be important for efficient establishment and linked to mature plant traits such as height and yield. A root phenotyping pipeline, consisting of a germination paper-based screen combined with image segmentation and analysis software, was developed and used to characterize seedling traits in 94 doubled haploid progeny derived from a cross between the winter wheat cultivars Rialto and Savannah. Field experiments were conducted to measure mature plant height, grain yield, and nitrogen (N) uptake in three sites over 2 years. In total, 29 quantitative trait loci (QTLs) for seedling root traits were identified. Two QTLs for grain yield and N uptake co-localize with root QTLs on chromosomes 2B and 7D, respectively. Of the 29 root QTLs identified, 11 were found to co-localize on 6D, with four of these achieving highly significant logarithm of odds scores (>20). These results suggest the presence of a major-effect gene regulating seedling root vigour/growth on chromosome 6D.

## Highlight

A phenotyping pipeline was used to quantify seedling root architectural traits in a wheat double haploid mapping population. QTL analyses revealed a potential major effect gene regulating seedling root vigour/growth.

## Introduction

Bread wheat (*Triticum aestivum* L.) is a crop of global importance accounting for ~20% of all calories consumed worldwide (Food and Agriculture Organization of the United Nations, 2013). Root system architecture (RSA, the spatial configuration of a root system in soil) is critical for nutrient and water uptake, anchorage, nutrient storage, and plant–microbe interactions. Thus RSA has a direct impact on grain yield (Lynch, 2007; Smith and Smet, 2012). However, breeding and selection programmes have not directly considered RSA to date, mainly due to the difficulty in observing root traits in soil (Waines and Ehdaie, 2007). This may have resulted in non-optimal root systems, as suggested by some ‘Green Revolution’ wheats having root biomass less than two-thirds the mean of some landraces (Waines and Ehdaie, 2007). The *Reduced height* (*Rht*) genes, which control shoot height in wheat and are present in many modern cultivars, have been reported to reduce root proliferation (Bai *et al.*, 2013). This has prompted the proposal that optimization of RSA should form the basis of a second Green Revolution to produce the increase in below-ground resource capture and yield required to meet the needs of the increasing global population (Lynch, 2007). Furthermore, optimization of RSA may be a promising avenue to enhance nitrogen (N) uptake efficiency and hence reduce N fertilizer requirements with associated environmental and economic benefits (Foulkes *et al.*, 2009).

Traditional methods for the analysis of root systems such as soil cores, soil columns and rhizotrons provide high levels of detail, but are labour intensive and time consuming, and are thus often unsuitable for the study of large mapping populations for quantitative trait locus (QTL) analyses (Blum and Arkin, 1984; Kuecke *et al.*, 1995; César de Carvalho, 2009). Researchers have therefore adopted various artificial media systems to study roots such as hydroponic culture, agarose gel chambers, and systems using germination paper (Zhu *et al.*, 2005; Hund *et al.*, 2009; Clark *et al.*, 2011; Liu *et al.*, 2013; Bai *et al.*, 2013; Sun *et al.*, 2013). Systems based on germination paper pouches were first developed for the study of basal root angle in *Phaseolus vulgaris* (Bonser *et al.*, 1996) and later adapted for the manual measurement of root length and angle in the same species (Liao *et al.*, 2001). This approach was successfully adapted for maize seedlings and combined with flat-bed image scanning to allow digital image analysis of RSA (Hund *et al.*, 2009). In recent years QTL detection based on high-density genetic maps has increased our understanding of the genetic control of root traits in cereals. Several studies have indicated overlaps between QTLs for nutrient uptake and root traits in wheat (An *et al.*, 2006), maize (Tuberosa *et al.*, 2002), and rice (Steele *et al.*, 2007). There have also been a considerable number of studies identifying QTLs for seedling root traits in wheat (Clark *et al.*, 2011; Hamada *et al.*, 2012; Bai *et al.*, 2013; Christopher *et al.*, 2013). Although there are obvious limitations to the study of seedling root traits, vigorous early root growth has been shown to be a major factor influencing N uptake (Liao *et al.*, 2004), and other seedling root traits such as the angle of seminal root emergence have been linked to root distribution over depth at later growth stages and hence drought tolerance (Manschadi *et al.*, 2007).

In this study, a high-throughput seedling phenotyping pipeline was developed to quantify wheat seedling RSA traits in the Savannah × Rialto doubled haploid (DH) mapping population, with the aim of identifying QTLs for seedling root traits related to early vigour and enhanced capacity for N uptake.

## Materials and methods

### Plant materials

The DH population was developed using the wheat-maize technique (Suenaga, 1994). A population of 94 DH lines was derived from the F_1_ between cultivars Savannah and Rialto. Savannah is a UK winter wheat semi-dwarf (Rht-D1b) cultivar first released in 1998; it is a hard endosperm cultivar with high yield potential used as feed wheat. Rialto is a UK winter wheat semi-dwarf (Rht-D1b) cultivar first released in 1995; it is a hard endosperm cultivar suitable for some bread-making processes.

### Field trials

Field experiments were carried out in 2007–8 and 2008–9 at two sites in the UK, at the University of Nottingham, Sutton Bonington (SB) (52°50 N, 1°14W) and at the John Innes Centre, Norwich (NO) (52°38 N, 1°18 E); and at one site in France, at INRA Estrées-Mons (EM) (49°08 N, 03°00 E). The experiment at SB used a split-plot design in which N treatment was randomized on main plots, genotypes were randomized on the sub-plots, and each treatment was replicated two times. Sub-plot size was 6×1.65 m. At NO and EM, the two N treatments were conducted in adjacent areas as randomized block designs. Plot size was 6×1.2 m at NO and 5×1.3 m at EM. The experiments were sown in early to mid-October at SB and NO and in mid- to late October at EM in each year. The soil type was a sandy loam at SB, a clay loam at NO, and a fine clay loam at EM. Different seed rates were used across experiments sufficient to establish a target of 200 plants m^–2^ in the spring and ranged from 250 to 350 seeds m^–2^.

In the high N treatment (HN), the amount of N applied varied from 200 to 250kg N ha^–1^ in the experiments. For each HN treatment, N was applied using either three (SB and NO) or four (EM) split applications. In each experiment, an initial application of 34–60kg N ha^−1^ was applied in March. In the experiments at SB and NO, the remainder was applied split approximately two-thirds at the start of stem extension [growth stage (GS) 31 (Zadoks *et al.*, 1974)] and one-third at flag leaf emergence (GS39). In the experiments at EM the remainder was applied split approximately two-fifths at GS31, two-fifths at GS39, and one-fifth at anthesis (GS61). In the low N (LN) treatment, the amount of N applied varied in the experiments from 0 to 50kg N ha^–1^, depending on soil mineral content in February, and was applied at GS31. All N fertilizer N was applied as granules of ammonium nitrate (34.5% N) except for the first three applications in EM, which were applied as a liquid solution (15% ureic acid, 7.5% ammonium, 7.5% nitrate). Each split was applied on the same calendar date for each genotype. All other crop inputs, including weed, disease, and pest control, and potassium, phosphate, and sulphur fertilizers, were applied at levels to prevent non-N nutrients or weeds, diseases, and pests from limiting grain yield. Plant growth regulator was applied as chlormequat at onset of stem extension (GS31) in the experiments.

In all plots in all experiments, a small plot combine was used to determine the grain yield per unit of ground surface area (GY) from an individual plot area of at least 5 m^2^, and values adjusted to 0% moisture at harvest (GS92). Mature plant height (HT) was measured from soil level to the collar of each wheat ear; the mean of two measurements was taken for each plot. Before machine harvesting, in all plots of all experiments, crop dry mass and N content were assessed on a random sample of ~100 ear-bearing shoots removed from each plot by cutting at ground level. The number of fertile shoots (those with an ear) in each sample was counted. The weights of the straw, chaff, and grain (after threshing the ears) were recorded, after drying at 80°C for 48h. The concentration of N in the straw, chaff, and grain was measured using the Dumas method. Using these data, estimates of the above-ground N per unit area were made (NUp).

### Root system phenotyping

**Fig. 1.**
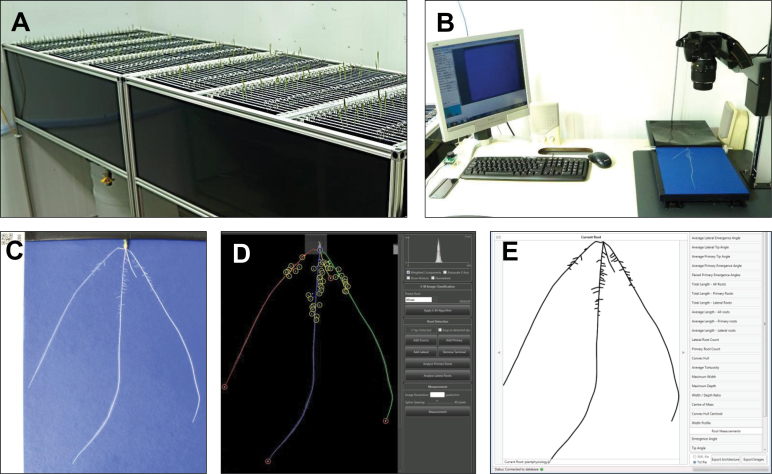
Phenotyping pipeline. (A) Growth assembly. (B) Image acquisition. (C) Example root image. (D) Root system extraction and quantification using RootNav software. (E) Reconstruction of root system *in silico* and trait measurement.

Seeds were sieved through a set of calibrated graduated sieves (Scientific Laboratory Supplies Ltd, Hessle, UK). All seeds used in the experiment were taken from the fraction collected between 2.8mm and 3.35mm mesh sizes. This size was selected as it represented the average seed size of both parental lines. Seeds were surface sterilized by rinsing in 70% (v/v) ethanol for 30 s, followed by transfer to 5% (v/v) sodium hypochlorite solution for 10min, and finally washing three times with sterile water. Sterilized seeds were placed onto moistened germination paper crease-side down and incubated at 4°C for 5 days to synchronize germination. Following cold treatment, seeds were transferred to a light-impermeable box for 48h to complete germination. The box was placed inside the controlled environment room where subsequent phenotyping was conducted (12h photoperiod: 20°C day, 15°C night, with a light intensity of 400 µmol m^–2^ s^–1^ PAR). Uniformly germinated seeds with roots ~5mm in length were transferred to individual growth pouches. The growth pouch system was based on the design developed for maize (Hund *et al.*, 2009). Each pouch consisted of a sheet of germination paper (24×30cm; Anchor Paper Company, St Paul, MN, USA), covered with a black polythene film of equal area (75 µm thick; Cransford Polythene Ltd, Suffolk, UK). The germination paper and film were fixed to an acrylic rod (316×15×5mm; Acrylic Online, Hull, UK) using two 18mm foldback clips. A matrix barcode label affixed to the rod allowed identification of each seedling. A single seedling was placed in each pouch centred 2cm from the top edge and held in place by the adhesion of the polythene sheet to the wet germination paper. Growth pouches were fitted into four aluminium and polypropylene frame assemblies (Fig. 1A) in the controlled environment chamber. Each assembly consisted of an aluminium profile frame (104×62×102cm; KJN Ltd, Leicester, UK) supporting toothed acrylic holders to suspend each pouch in a set position. Black polypropylene side panels (101×31×0.3cm and 63×31×0.3cm; Cut Plastic Sheeting, Devon, UK) maintain the pouches in darkness.

The base of each frame held a black polypropylene tray (99×61×10cm; Stansa Plastic Fabrication Ltd, Suffolk, UK) containing 18 l modified one-quarter Hoagland’s solution (Hoagland and Arnon, 1950) with HEDTA as the iron chelator (Piñeros *et al.*, 2005). The composition (mg l^–1^) of the nutrient solution was: (NH_4_)_3_PO_4_, 29mg; Ca(NO_3_)_2_, 165mg; MgSO_4_, 251.87mg; KNO_3_, 151.99mg; H_3_BO_3_, 28.44mg; Cu_2_SO_4_, 75mg; MnCl_2_(H_2_O)_4_, 10.1mg; MoO_3_, 0.2mg; ZnSO_4_, 2.29mg; FeHEDTA, 25.49mg. The solution was adjusted to pH 6 using KOH. The volume of nutrient solution in each tray was maintained automatically via a float valve system and header tank containing deionized water. Each frame assembly consisted of three rows of 30 pouches allowing 90 plants per frame. Pouches were suspended so that the bottom 3cm of the pouch was submerged in the nutrient solution.

A randomized block design was used (each table acting as a block). Eighteen lines were germinated for each experiment, with a minimum target of 15 seedlings per line. Phenotyping of the whole mapping population was split over six experiments. After 9 days (two-leaf stage), individual pouches were transferred to a copy stand (model number SGCS-920; Speed Graphic, Hampshire, UK) for imaging using a Nikon D600 DSLR camera (Fig. 1B) controlled using NKRemote software (Breeze Systems Ltd, Camberley, UK). The copy stand was modified with two draw slides (RS UK, Northants, UK), a Nylatron block (600×260×22mm), and white acrylic sheets (330×290×9mm and 290×290×9mm; Cut Plastic Sheeting, Devon, UK) to form a template to ensure consistent placement of each pouch. The polythene film covering each pouch was carefully peeled back leaving the roots fixed to the blotting paper for imaging. The draw slides then enabled the template block to be repositioned allowing shoots to be imaged without moving the pouch. Images were taken of 8–36 seedlings of 92 lines of the mapping population together with the parental lines, resulting in 1709 RSA images.

### Image analysis

Root system images were processed using RootNav software (Pound *et al.*, 2013). Traits were quantified from extracted RSA using RootNav’s standard functions (Table 1) as detailed in Pound *et al.* 2013. For this study, RootNav functionality was extended to measure additional traits describing the distribution of the root system (the co-ordinates of the centre of mass of the root system and the co-ordinates of the centroid of the convex hull), determined as follows.

**Table 1.** Root system architectural traits

Acronym	Description (units)
RTLA	Total length of all roots (mm)
RTLS	Total length of seminal roots (mm)
RTLL	Total length of lateral roots (mm)
RSC	Number of seminal roots
RLC	Number of lateral roots
RMW	Maximum width of the root system (mm)
RMD	Maximum depth of the root system (mm)
RMWD	Width-depth ratio (RMW/RMD)
RCMX	Centre of mass of the root system, horizontal co-ordinate (mm)
RCMY	Centre of mass of the root system, vertical co-ordinate (mm)
RCH	Convex hull, area of the smallest convex polygon to enclose the root system (mm^2^)
RCHCX	Centre of mass of the convex hull, horizontal co-ordinate (mm)
RCHCY	Centre of mass of the convex hull, vertical co-ordinate (mm)
RAE1	Angle of emergence between the outermost seminal roots measured at 30 *px* (degrees)
RAE2	Angle of emergence between the second pair of seminal roots measured at 30 *px* (degrees)
RAE951	Angle of emergence between the outermost seminal roots measured at 95 *px* (degrees)
RAE952	Angle of emergence between the second pair of seminal roots measured at 95 *px* (degrees)
RAE251	Angle of emergence between the outermost seminal roots measured at the first quartile of the total length (degrees)
RAE252	Angle of emergence between the second pair of seminal roots measured at the first quartile of the total length (degrees)
RAE501	Angle of emergence between the outermost seminal roots measured at the second quartile of the total length (degrees)
RAE502	Angle of emergence between the second pair of seminal roots measured at the first quartile of the total length (degrees)
RAE751	Angle of emergence between the outermost seminal roots measured at the third quartile of the total length (degrees)
RAE752	Angle of emergence between the second pair of seminal roots measured at the third quartile of the total length (degrees)
RAE1001	Angle of emergence between the outermost seminal roots measured at the root tip (degrees)
RAE1002	Angle of emergence between the second pair of seminal roots measured at root tip (degrees)

#### Centre of mass of root system 

The centre of mass for each root system was calculated under the assumption that the density of root material is evenly distributed along the length of each root. Points were sampled regularly, at one pixel intervals along each root. The 2D centre of mass, R, was calculated as:

$$\text{R}=\frac{1}{\text{n }\sum_{\text{i}=1}^{\text{n}}\left|{\text{S}}_{i}\right|}\;\overset{\text{n}}{\underset{\text{i}=1}{\sum }}\overset{\left\lfloor {\text{S}}_{i}\right\rfloor }{\underset{\text{t}=0}{\sum }}{\text{S}}_{i}\left(\text{t}\right),$$ (1)

where *n* is the number of roots, *S*_*i*_ is the geometry of a given root *i*, and *S*_*i*_(*t*)is the 2D position of the root at distance t along its length.

#### Centroid of convex hull 

The convex hull of the root system is calculated using the standard definition, that of the smallest convex set of *X* points containing the entire root system. This results in a set of points (*x*_1_, *y*_1_), ..., (*x*_n_, *y*_n_), the centroid (geometric centre) of which is calculated as:

$${\text{C}}_{x}=\frac{1}{6\text{A}}\overset{\text{n}}{\underset{\text{i}=0}{\sum }}({\text{x}}_{i}+{\text{x}}_{\text{i}+1})({\text{x}}_{i}{\text{ y}}_{\text{i}+1}-{\text{x}}_{\text{i}+1}{\text{ y}}_{\text{i}})$$ (2)

$${\text{C}}_{y}=\frac{1}{6\text{A}}\overset{\text{n}}{\underset{\text{i}=0}{\sum }}\left({\text{y}}_{i}+{\text{y}}_{\text{i}+1}\right)\left({\text{x}}_{i}\;{\text{y}}_{\text{i}+1}-{\text{x}}_{\text{i}+1}\;{\text{y}}_{i}\right),$$ (3)

where A is the signed area of the convex hull,

$$\text{A}=\frac{1}{2}\overset{\text{n}}{\underset{\text{i}=0}{\sum }}\left({\text{x}}_{i}\;{\text{y}}_{\text{i}+1}-{\text{x}}_{\text{i}+1}\;{\text{y}}_{i}\right)$$ (4)

It should be noted that the point (*x*_*n*+1_, *y*_*n*+1_) is equal to (*x*_1_, *y*_1_)in the above formulae.

**Fig. 2.**
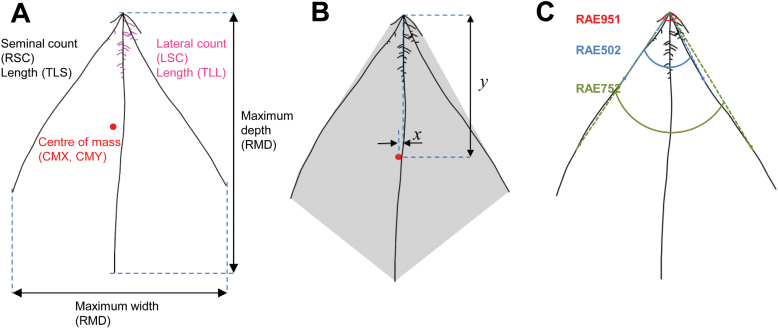
(A) Seedling traits. (B) Convex hull (the smallest area that encloses the whole root system, shown in grey). The centroid (geometric centre) of the convex hull is marked by the red circle and its vertical and horizontal coordinates relative to the seed as *y* and *x*, respectively. (C) Root angle measurements. RAE951: angle between paired outer roots (at 15mm from the seed). RAE502 and RAE752: angle between inner pair of roots (at 50 and 75% of root length, respectively).

As root angle varies with position along the root and the angle between pairs of seminal roots has been shown to be significant (Christopher *et al.*, 2013), additional angle measurements were calculated at a fixed point 15mm along each root, and at 50, 75, and 100% of root length (Fig. 2C).

### QTL analysis

Statistical analysis was performed using the R software suite (vs 3.0.2) (R Core Team, 2014). ANOVA was applied to the raw data for each trait and showed significant differences between genotypes. For some of the root trait measurements significant differences between experiments were found (RAE1, RAE1002, RCH, RCHCY, RCMY, RLC, RMD, RMW, RMWD, RTLA, RTLL, and RTLS; Table 1). In those cases a fixed term for the experiment was included in the model and best linear predictors (BLUPs) were calculated, so that final trait values were corrected for these differences. For all other trait values best linear estimators (BLUEs) were calculated (RAE2, RAE251, RAE252, RAE501, RAE502, RAE751, RAE752, RAE951, RAE952, RAE1001, RCHCX, RCMX, RSC, and field traits). Spearman rank correlation coefficients (*ρ*) between traits and tests for the association being different from zero were calculated on BLUEs or BLUPs,

QTL calculation and plotting of logarithm of odds (LOD) scores were conducted using R package ‘qtl’ on BLUEs or BLUPs, in the first step as a single QTL model employing the extended Haley-Knott method on imputed genotypes. Significant thresholds for the QTLs were calculated from the data distribution. Final QTL LOD scores and effects were received from a multiple QTL model, using the QTL detected in the initial scan. The high-density Savannah × Rialto iSelect map (Wang *et al.*, 2014) was used, with redundant markers and those markers closer than 0.5 cM stripped out. For HN, GY, and NUp, only QTL locations where a root QTL was identified on the same chromosome are listed in detail.

## Results

### Phenotyping pipeline

The workflow of the phenotyping pipeline is shown in Fig. 1. Seedlings from 92 lines of the Savannah × Rialto DH mapping population were grown together with the parental lines in growth pouches in a controlled environment chamber (Fig. 1A; Materials and Methods). The root and shoots of a total of 1709 seedlings were imaged after 9 days. Root images were processed using RootNav software (Pound *et al.*, 2013) to extract RSA (Fig. 1C–E). A total of 25 traits were quantified from the extracted root architectures (see Table 1, Fig. 2). Measurements of root counts, length, emergence angles, and convex hull were determined using native functions in RootNav (see Fig. 2). Additional measurements of the spatial co-ordinates of the centre of mass of the root system and the centroid of the convex hull and seminal root angles were determined as described in the Materials and Methods.

### Root phenotypic variation between parent and DH lines

**Fig. 3.**
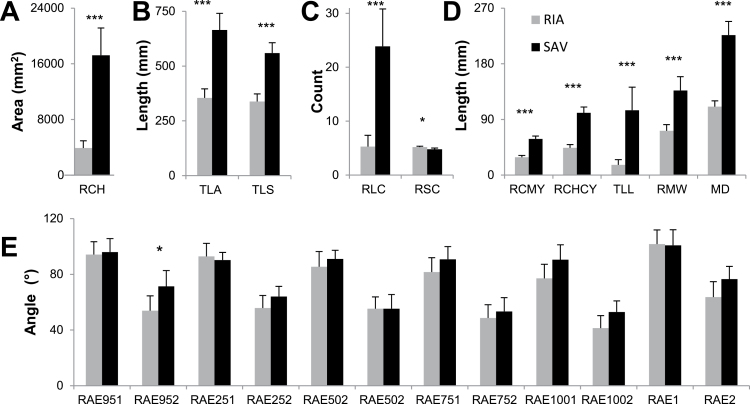
Phenotypic variation in parental line root traits (*n* = 18 for Savannah; *n* = 22 for Rialto). (A) Area of convex hull (RCH). (B) Length of total root system (TLA) and seminal roots (TLS). (C) Number of lateral (RLC) and seminal (RSC) roots. (D) Vertical co-ordinates of centre of mass (RCMY) and convex hull (RCHCY); total length of lateral roots (TLL); maximum horizontal width of root system (RMW); maximum depth of root system (MD). (E) Angle measurements. Error bars are two standard errors of the means; *, *P* < 0.05; ***, *P* < 0.001.

**Fig. 4.**
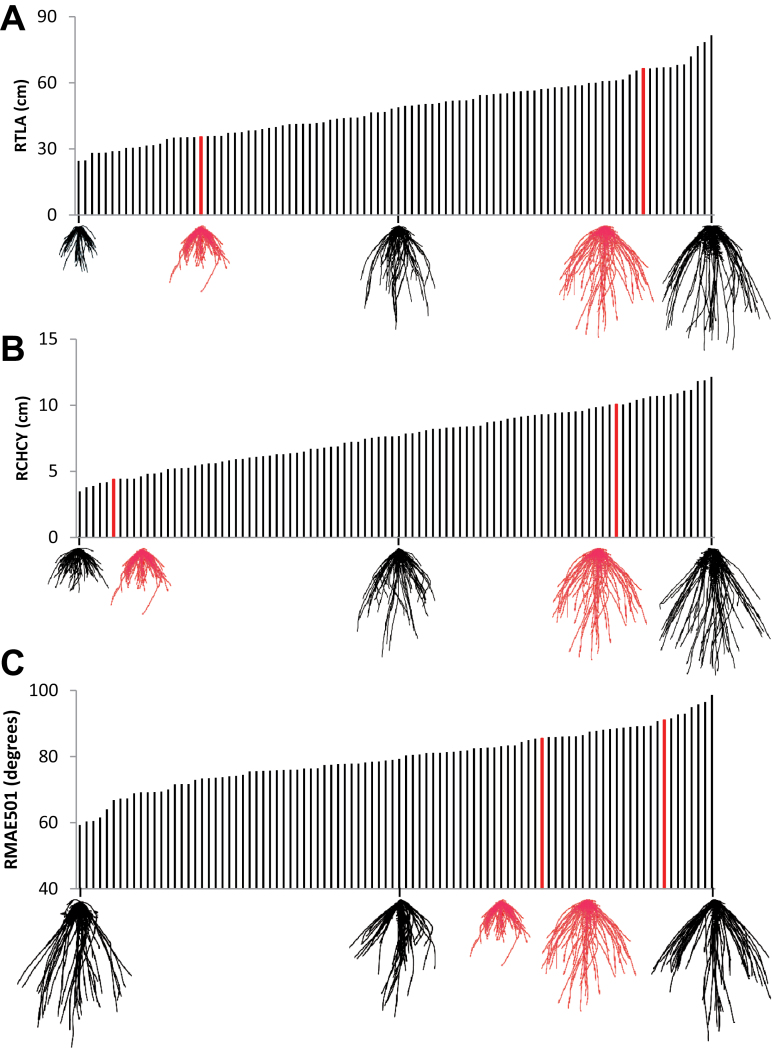
Phenotypic variation in RSA traits. Images are overlays of RSA images with parental lines shown in red (*n* = 18 for Savannah, right; *n* = 22 for Rialto, left). (A) Total root length. (B) y co-ordinate of convex hull centroid. (C) angle between outer pair of seminal roots at 50% seminal root length. For B-D, values are ordered by increasing value. Images are overlays of RSA images (*n* = 14 to 19) for the extreme and median values. The parental line values are shown in red.

The two parental lines, Savannah and Rialto, showed significant differences (*P* < 0.05 and lower) between all measured root traits except angle traits (Fig. 3). Mean lengths of seminal and lateral roots were 60 and 15.7% longer, respectively, in Savannah compared to Rialto. The mean number of seminal roots in Savannah was less than that in Rialto (by 8.7%); for lateral roots, the opposite was observed, with Savannah on average having 22% more laterals. Savannah had a 22.7% larger convex hull (CH), a 52.3% greater maximum width (MW), and a 48.9% greater maximum depth (MD). In contrast, of the angle traits recorded, only the second seminal angle at 15mm (RAE952), showed a significant difference of 17.4° (*P* < 0.05) between the parental mean values. Lines within the DH population showed transgressive segregation (values more extreme than the parental phenotypes) for all root traits scored (Fig. 4 and Supplementary Table S1). Fig. 4A–C shows the mean values for the DH population for three traits with overlay images of the extracted RSA for the lines exhibiting extreme and median values. Overlay images of the parental lines are shown in red. Frequency histograms for the measured root traits are given in Supplementary Figure S1.

### Correlations between seedling traits

The traits RAE2, RAE251, RAE252, RAE501, RAE502, RAE751, RAE752, RAE951, RAE952, RAE1, RAE1001, RAE1002, and partly RMWD showed significant and highly positive correlation amongst DH lines (*ρ* between 0.7 and 0.99; *P* < 0.05; Supplementary Table S2).

Similarly, traits RLC, RCH, RMD, RWM, RCMY, RCHCY, RTLA, RTLL, and RTLS were significantly and highly positively correlated amongst DH lines (*ρ* between 0.7 and 0.99; *P* < 0.05). Several traits showed a negative relationship to one another between these groups.

Trait RSC (number of seminal roots) showed some neutral and slight negative relationships to both groups and was not strongly correlated to any of the root traits. Traits RCHCX and RCMX showed a strong correlation to one another, but not to any other of the root traits.

The root traits can be assigned to five categories following the association between them: (i) the root angle traits; (ii) the root length (irrespective of root category), co-ordinates of the centre of the area measurements in the *y*-axis, lateral root count, and RCH, RMW, and RMD; (iii) co-ordinates of the centre of the area measurements in the *x*-axis; (iv) seminal root count (RSC); and (v) the maximum root width to depth ratio.

### Field traits

Average GY across the sites and years ranged between 7.5 and 12.2 t ha^–1^ under HN and 5.3 and 8.0 t ha^–1^ under LN, with yields in EM at the lower end. Average HT values were 69.2–85.7cm under HN and 52.5–76.4cm under LN with NO plants being the shortest. Average NUp values were 27.7–32.4g m^–2^ under HN and 8.4–23.7g m^–2^ under LN, with EM values being lowest under HN (see Supplementary Figure 2).

The correlations between root traits and GY were not significant in the majority of trials (88%). However, some trends can be seen in that correlations under HN are more likely to be significant (18% of HN trials), and correlations between GY and RCH, RCHCX, RCHCY, RCMX, RCMY, RMD, RMWD, RTLA, and RTLS are more likely to be significant than the other root traits (*ρ* generally low, between –0.34 and 0.39). Correlation trends between root traits and HT have a similar pattern to those with GY, but are even less frequently significant (*ρ* generally low, between –0.25 and 0.29). Correlation trends between root traits and NUp were not significant under LN and no particular pattern of consistent correlations could be observed under HN (*ρ* generally low, between –0.24 and 0.28)).

### QTL detection

For all root traits measured, 29 QTLs were discovered, and these were located on chromosomes 1A, 2B, 3B (four QTLs), 3D (three QTLs), 4D, 6D (10 QTLs), 7A, and 7D (seven QTLs). A more detailed list is given in Table 2.

**Table 2.** QTLs identified

Trait	Chr	Pos	LOD	%var	add eff	+	Peak marker	CI begin	CI end	Env
NUp	1A	106	3.9	16.3	–0.5	Sav	wsnp_JD_c6544_7697235	87.5	221.3	SBLN8
RCMX	1A	70.3	2.5	8.8	–0.8	Sav	GENE-0509_78	47.7	163.6	
RRAE502	2B	77	2.9	13.8	3.5	Ria	Tdurum_contig42153_4175	54.1	114.4	
HT	2B	82	4.4	10.4	–1.9	Sav	wsnp_Ku_ c34759_44069854	54.1	149.4	NOLN8
RAE2	3B	178.8	3.8	15.5	–5.2	Sav	RAC875_c59977_598	161.8	190.8	
RRAE251	3B	178.8	3.5	16.6	–4.7	Sav	RAC875_c59977_598	154.5	182.7	
RRAE951	3B	178.8	2.7	12.8	–5.1	Sav	RAC875_c59977_598	148	296.4	
RRAE952	3B	178.8	3.5	14.4	–4.7	Sav	RAC875_c59977_598	154.5	192.4	
RRAE252	3D	5.8	2.9	13.8	–3.7	Sav	BS00004334	0	36	
RRAE952	3D	5.8	3.7	15.1	–4.9	Sav	BS00004334	0	36	
RAE2	3D	5.8	3.6	14.5	–4.9	Sav	BS00004334	0	36	
RMWD	4D	4.8	2.7	13.2	0.0	Ria	wsnp_Ex_c9440_15657149	0.8	67.6	
GY	6D	125.1	2.5	10.3	–0.2	Sav	RAC875_c9618_334	53	144	EMHN8
RCHCY	6D	4.4	22.4	68.6	–17.1	Sav	wsnp_Ex_c4789_8550135	2	53	
RTLA	6D	4.4	24	58.7	–100.8	Sav	wsnp_Ex_c4789_8550135	2	53	
RTLS	6D	4.4	25.6	60.9	–84.5	Sav	wsnp_Ex_c4789_8550135	2	53	
RCH	6D	4.4	17.6	52.8	–3593	Sav	wsnp_Ex_c4789_8550135	2	53	
RCMX	6D	26	2.8	10.1	0.7	Ria	wsnp_Ex_c4789_8550135	0	92.5	
RCMY	6D	4.4	19.1	62.8	–9.7	Sav	wsnp_Ex_c4789_8550135	2	53	
RLC	6D	4.4	9.1	33.7	–4.2	Sav	wsnp_Ex_c4789_8550135	0	53	
RMD	6D	4.4	22.7	61	–30.1	Sav	wsnp_Ex_c4789_8550135	2	53	
RMW	6D	4.4	6.4	28.3	–16.1	Sav	wsnp_Ex_c4789_8550135	0	53	
RTLL	6D	4.4	6.4	25.2	–16.6	Sav	wsnp_Ex_c4789_8550135	0	53	
GY	7A	64	3.5	13.8	0.3	Ria	BS00009677_51	49.3	151.5	NOHN8
RSC	7A	14.4	2.1	10.2	0.1	Ria	EXcalibur_c8522_1894	0	40.2	
GY	7D	16	3.1	12.1	–0.2	Sav	wsnp_Ku_c416_869895	16	38.8	NOLN9
RCHCX	7D	22	3	14.5	1.2	Ria	wsnp_Ra_ c8297_14095831	16	101.8	
GY	7D	16	2.2	8.8	–0.2	Sav	wsnp_Ku_c416_869895	16	38.8	EMLN9
GY	7D	16	4	18.4	–0.3	Sav	wsnp_Ku_c416_869895	16	38.8	NOHN9
NUp	7D	20	4.7	13.9	–0.5	Sav	wsnp_Ra_ c8297_14095831	16	62.4	EMHN8
RCMX	7D	19	2.7	9.7	0.9	Ria	wsnp_Ra_ c8297_14095831	16	38.8	
RTLA	7D	27	9	14.2	–53.5	Sav	wsnp_Ra_ c8297_14095831	16	52	
RTLS	7D	27	9.7	14.5	–44.5	Sav	wsnp_Ra_ c8297_14095831	16	52	
RLC	7D	29	2.4	7.5	–2.4	Sav	Kukri_c48125_714	16	101.8	
RMD	7D	30	5.8	9.6	–13.3	Sav	Kukri_c48125_714	16	52	
RCH	7D	34	3.5	7	–1702	Sav	Kukri_c48125_714	16	62.4	
RTLL	7D	31	2	6.9	–10.1	Sav	Kukri_c48125_714	16	101.8	

Chr, chromosome; pos, position; %var, percentage variance explained; add eff, additive effect (units as Table 1); +, positive allele coming from Sav (Savannah) or Ria (Rialto); CI, confidence interval; CI begin, start position of CI; CI end, end position of CI; env, environment. For field traits: NO, Norwich; EM, Estrées-Mons; SB, Sutton Bonington; HN, high-N treatment; LN, low-N treatment; number denotes year (8, 2007–8; 9, 2008–9).

The QTL on 1A for RCMX explained 10% of the variation observed; the 2B QTL for RAE502, 14%; the 3B QTLs for RAE2, RAE251, RAE951, and RAE952, 13–17%; the 3D QTLs for RAE2, RAE252, and RAE952, 14–15%; the 4D QTLs for RMWD, 15%; the 6D QTLs for RCH, RCHCY, RCMX-Y, RLC, RMW, RTLA, RTLL, and RTL5, 25–62%; the 7A QTL for RSC, 10%; and the 7D QTL for RCHCX, RCMX, RLC, RMD, RTLA, RTLL, and RTL5, 7–15%.

For HT, seven QTLs were detected, located on chromosomes 1B, 1D, 2A, 2B, 3A, 5A and 6A. Only the 3B HT QTL, with the positive effect coming from Rialto, co-locates with the RAE2 root trait QTL. Six GY QTLs were detected, located on chromosomes 1B, 2B, 3A, 6A, 7A, and 7D. The 7D GY QTL co-locates with several 7D root QTLs. For NUp, QTLs were detected on chromosomes 1A, 1B, 2A, 2D, 3A (three environments), 4B, 5A, 5D, and 7D. QTLs explained variation between 7.1 and 26.8%, with the biggest effect on 3A present only in low-N environments, with the allele with increasing effect coming from Rialto, in EMHN8, EMLN8, SBLN9, and NOLN8 (near significant). The 7D QTL, present in EMHN8 and possibly EMHN9, co-locates with the 7D root QTL. The increasing effect of the 7D root trait QTL, as is the case for the NUp QTL, comes in both cases from Savannah. For HT, QTLs were detected on chromosomes 1B, 1D, 2A, 2B, 3A, 5A, and 6A. Only the 3B QTL, with the positive effect coming from Rialto, co-locates with one of the root traits, RAE2. The GY QTLs were detected on chromosomes 1B, 2B, 3A, 6A, 7A, and 7D. The 7D QTL possibly co-locates with the 7D root QTL and the 7D NUp QTL.

## Discussion

In the present study, a high-throughput phenotyping pipeline was utilized to reveal a number of new QTLs for seedling root traits in a DH mapping population of hexaploid wheat. The pipeline allows for the detailed phenotyping of 360 plants (assuming four frame assemblies in a standard growth chamber) with an experimental duration of 2 weeks including image analysis, making it suitable for the phenotyping of large mapping populations for QTL studies. Image acquisition of 360 seedlings takes ~3h. User-supervised image analysis using RootNav takes on average 2min per image. A typical experiment thus requires 4 days of user time (2 days to assemble pouches and sterilize, germinate, and transfer seeds; 2 days for the image acquisition and analysis steps). This equates to ~5min per plant.

Although phenotyping systems based on germination paper have been described in previous studies (Zhu *et al.*, 2005; Hund *et al.*, 2009; Bai *et al.*, 2013), this pipeline adds several enhancements; these include the use of a fixed software-controlled digital camera and custom copy stand rather than the more commonly used scanner (reducing acquisition time whilst maintaining high image resolution) and the use of dedicated software for image segmentation and subsequent trait quantification. RootNav stores the raw architecture of each root system, instead of recording specific trait measurements. Traits are then quantified by querying a database of stored architectures for the trait(s) of interest. The advantage of this approach is that new traits can be added and quantified without the need to re-analyse the original images. For this study, several new traits were added to the standard RootNav functions: the co-ordinates of the centre of mass and the centroid of the convex hull, and angles between paired seminal roots. Highly significant QTLs were identified for both trait classes (see Table 2). A further advantage of this approach is that the stored architectures can be exported in standard formats for analysis using other software packages if desired. To this end, RootNav can export data in raw tabular form or in the Root System Markup Language (RSML) standard (Lobet et al., 2015).

### Root QTLs

**Fig. 5.**
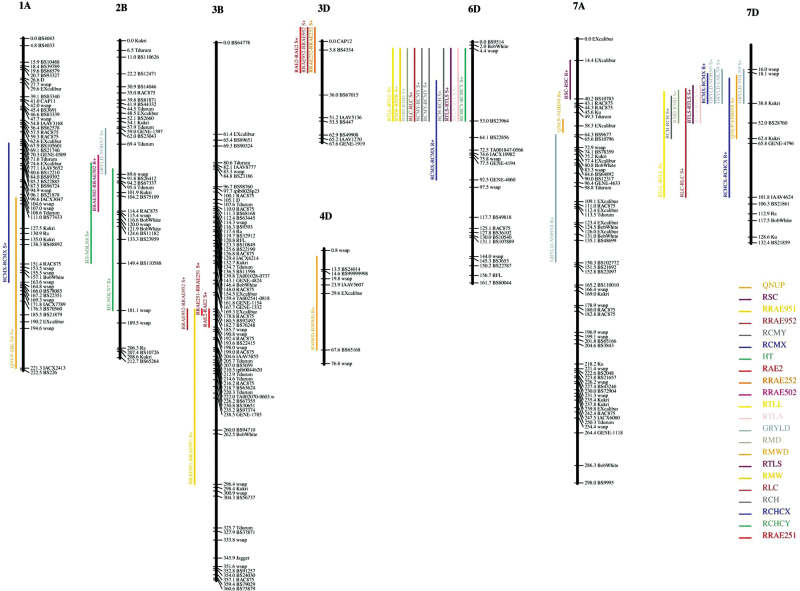
Diagrams of eight wheat chromosomes (1A, 2B, 3B, 3D, 4D, 6D, 7A, 7D) with QTL locations. Chromosomes are depicted in black; a selection of markers from the iSelect Savannah × Rialto map and their mapping positions are shown to the right of the chromosomes. Only chromosomes that carry a seedling root QTL are shown. QTL confidence intervals are shown as vertical bars to the left of the chromosomes; bar colours are specific for traits as defined in the key. Trait names and environments are shown above each bar. S+, positive effect comes from Savannah; R+, positive effect from Rialto.

In total, 29 root QTLs were discovered on chromosomes 1A, 2B, 3B, 3D, 4D, 6D, 7A, and 7D (Table 2, Fig. 5). In all root length-type traits, Savannah contributed the positive alleles leading to a larger root system. This is consistent with the observation that Savannah has a larger root system during early seedling growth stages (Fig. 3 and Fig. 4).

The correlation analysis (Supplementary Table S2) allows the root architectural trait data to be subdivided into five classes. QTLs for the first class, root angle traits, were limited to chromosomes 2B, 3B, and 3D, and did not co-localize with any other root traits (Supplementary Table S2). Interestingly, only one QTL (RCMX) was found within the A genome.

The second class of traits includes measurements of root lengths and width and distribution of the root system. Highly significant QTLs for this class were found on chromosome 6D, with traits RCHCY, RTLA, RTLS, and RMD achieving LOD scores > 20 (Table 2). In total, 11 significant root QTLs co-localized in this region, with traits representing measures of overall root system size, length, or root count. These results suggest the presence of a major gene regulating seedling root architecture and/or vigour in this region. Other studies have indicated root trait QTLs such as RTL and RSC on chromosome 6D, but not in the same region and with lower LOD values (Zhang *et al.*, 2013).

Values for the third class of traits, co-ordinates of the centre of the area measurements on the *x*-axis, vary by only 11mm for RCMX and 16mm for RCHCX across the mapping population. As these distances are relatively small, they are likely to be of little biological relevance, although significant QTLs for RCMX and RCHCX were identified on chromosomes 1A, 6D, and 7D.

The fourth class, seminal root count (RSC), is linked to a QTL on chromosome 7A (LOD 2.1) with the positive allele coming from Rialto.

The fifth class, the ratio between the maximum width and depth of the root system (RWMD) links to a QTL, separate from the other length and width QTLs, on chromosome 4D (LOD 2.7). Seedling root QTLs have been identified close to this location in another study using different germplasm, which also uncovered a co-localizing QTL for thousand grain weight (Bai *et al.*, 2013).

### Correlations between field N uptake and root data

Root traits did not strongly or consistently correlate with HT, NUp, or grain yield. One NUp QTL (LOD 4.7) on 7D co-localized with QTLs for RTLA and RTLS (LOD 9 and 9.7, respectively), potentially suggesting the same gene could be responsible for these traits. Other studies have also found limited correlation between HT and seedling root traits, but did find co-localizing QTLs (Bai *et al.*, 2013). QTLs for RAE502 and GY also co-localized to the same area of 2D (LOD 2.9 and 2.1, respectively) suggesting seedling root angle may affect field yield, or that genes in this region have an effect on both traits. Interestingly, a larger proportion of seedling root trait QTLs was detected on the D genome (24 QTLs on four chromosomes) than on the other two genomes (six QTLs on three chromosomes). This is quite different to the trend seen in HT and GRYLD, where only one of seven HT QTLs and one of six GRYLD QTLs came from the D genome. From this data it appears that the contribution of the D genome to development of the seedling root system is particularly important.

A limitation with seedling screens is that the results may not correlate to mature plant measurements taken in the field. A variety of factors may be responsible, such as the lack of adventitious roots at seedling growth stages, that account for a significant proportion of the final root system and are critical for nutrient and water uptake (Boatwright and Ferguson, 1967). Other factors such as seed size and endosperm composition are known to influence seedling growth (Maydup *et al.*, 2012), although in this study seeds were sieved to remove any size effect. The lack of consistent correlations between root traits and NUp is partly due to a strong genotype × N × site-year effect, but also by a high noise level, given that the trait is not directly measured but calculated from measurements taken from different organs.

In summary, a root phenotyping pipeline, consisting of a germination paper-based screen combined with image segmentation and analysis software, has been used to characterize root seedling traits in 94 lines of a DH population. In total, 29 QTLs for seedling root traits were identified. Two QTLs for traits quantified from field trials, grain yield and N uptake, co-localize with root QTLs on chromosomes 2B and 7D, respectively. Of the root QTLs identified, 11 were found to co-localize on 6D, with four of these achieving highly significant LOD scores (>20). These results suggest the presence of a major-effect gene regulating seedling root architecture and/or vigour in this region.

## Supplementary material

Supplementary data can be found at *JXB* online.

Supplementary Table 1. Root system trait values.

Supplementary Table 2. Correlation matrix for measured traits.

Supplementary Figure 1. Frequency distributions of measured root traits.

Supplementary Figure 2. Distribution of field traits.

## Funding

This work was supported by Biotechnology and Biological Sciences Research Council and Engineering and Physical Sciences Research Council Centre for Integrative Systems Biology programme funding to the Centre for Plant Integrative Biology; European Research Council Advanced Grant funding (FUTUREROOTS) to MJB, JA, MG, and DMW; BBSRC Professorial Fellowship funding to MJB and JA; Belgian Science Policy Office (grant IAP7/29) funding to DMW and MJB; a Royal Society Wolfson Research Merit Award to MJB; and Institut National de la Recherche Agronomique funding to JLG.

## Supplementary Material

Supplementary Data

## Abbreviations:

DH: doubled haploid
LOD: logarithm of odds
QTL: quantitative trait locus
RSA: root system architecture.
