# Supplementary material for: Phenotyping pipeline reveals major seedling root growth QTL in hexaploid wheat
Source: J Exp Bot. 2015 Mar 4;66(8):2283–92. doi: 10.1093/jxb/erv006 (PMC4407652; doi:10.1093/jxb/erv006)
Supplement: Supplementary Data [file supp_66_8_2283__index.html]

Phenotyping pipeline reveals major seedling root growth QTL in hexaploid wheat — Phenotyping pipeline reveals major seedling root growth QTL in hexaploid wheat — Supplementary Data 

# Phenotyping pipeline reveals major seedling root growth QTL in hexaploid wheat

## Supplementary Data

Data files

**Files in this Data Supplement:**

- Supplementary Data - Supplementary Data
