# Supplementary material for: Phenotyping pipeline reveals major seedling root growth QTL in hexaploid wheat
Source: J Exp Bot. 2015 Mar 4;66(8):2283–92. doi: 10.1093/jxb/erv006 (PMC4407652; doi:10.1093/jxb/erv006)

Novel phenotyping pipeline reveals major seedling root growth QTL in hexaploid wheat

Jonathan A. Atkinson, Luzie U. Wingen, Marcus Griffiths, Michael P Pound, Oorbessy Gaju, John Foulkes, Jacques Le Gouis, Simon Griffiths, Malcolm J. Bennett, Julie King, and Darren M. Wells

SUPPLEMENTARY MATERIAL

Supplementary Table 1. Root system trait values

Supplementary Table 2. Correlation matrix for measured traits

Supplementary Figure 1. Frequency distribution of measured root traits

Supplementary Figure 2. Distribution of field traits

Supplementary Table 1. Mean root system traits (RIA = Rialto, SAV = Savannah)

Trait	RIA	SD	Min	Max	SAV	SD	Min	Max	DH	SD	Min	Max
TLA (mm)	354.1	98.8	197	676	664.2	162	362.2	995.6	483.5	181.2	118.0	1191.1
TLL (mm)	16.4	20.5	0.0	92.3	105.0	78.9	1.2	323.4	32.6	43.4	0.0	340.9
TLS (mm)	337.7	82.5	195.5	583.7	559.2	100	360.9	733.9	450.9	151.7	118.0	1033.8
RAE1 (°)	101.7	21.5	56.2	141.78	100.8	19.5	60.1	142.3	93.6	29.9	5.0	203.7
RAE2 (°)	63.6	23.7	32.0	106.11	76.5	15.8	48.3	101.7	63.5	23.1	8.3	143.0
RLC	5.3	4.9	0.0	18	23.9	14.7	1.0	56.0	9.4	10.5	0.0	62.0
RSC	5.2	0.4	5.0	6	4.8	0.5	4.0	6.0	4.7	0.7	2.0	7.0
RCH (mm^2)	3910	2444	1170	12350	17243	8283	5281	35350	9152	6823	402	39353
RMW (mm)	71.6	23.7	21.5	122.9	136.8	47.5	50.8	212.5	89.2	43.0	5.7	239.1
MD (mm)	110.8	22.1	80.0	185.5	226.3	46.9	130.1	298.1	166.3	54.1	49.7	310.3
MWD	0.7	0.2	0.2	0.95	0.6	0.2	0.2	1.0	0.6	0.2	0.0	1.5
RCMX (mm)	0.2	3.4	-5.8	7.8	-0.5	5.8	-10.6	8.2	-1.9	6.4	-37.3	30.4
RCMY (mm)	28.9	6.7	17.5	48.3	58.5	10.2	36.7	78.2	50.6	17.5	11.5	100.9
RCHCX (mm)	0.9	5.2	-9.9	15.3	-2.8	15.2	-33.7	24.8	-4.1	9.7	-52.2	28.6
RCHCY (mm)	44.0	12.5	26.9	85.3	100.7	20.2	57.8	136.5	76.9	28.9	15.8	159.2
RAE951 (°)	94.1	19.7	48.3	117.08	96.0	16.6	67.4	131.6	88.2	22.2	0.4	185.5
RAE952 (°)	53.9	22.5	26.0	97.27	71.3	19.7	46.4	118.6	59.0	21.6	5.8	128.7
RAE251 (°)	92.9	19.9	44.6	115.99	90.3	9.3	66.6	102.9	84.4	20.8	6.6	161.1
RAE252 (°)	55.8	19.1	28.9	92.75	64.0	12.7	46.4	85.5	56.4	20.2	2.8	115.6
RAE502 (°)	85.4	23.3	25.2	114.24	91.0	10.8	65.7	104.4	79.6	20.8	5.1	140.6
RAE502 (°)	55.3	17.9	23.4	82.42	55.3	17.7	24.9	80.3	50.6	19.5	0.5	102.8
RAE751 (°)	81.6	22.0	26.1	110.28	90.8	15.7	59.3	116.4	76.9	21.3	2.4	168.1
RAE752 (°)	48.7	20.1	4.9	74.12	53.4	17.1	24.5	74.9	47.3	18.7	1.6	101.1
RAE1001 (°)	77.1	21.6	27.4	110.09	90.5	18.6	57.2	123.9	74.7	22.3	4.9	185.5
RAE1002 (°)	41.4	19.0	1.9	69.14	52.9	13.8	30.5	71.5	43.9	17.8	3.3	96.7

Supplementary Table 2. Correlation matrix for all measured traits

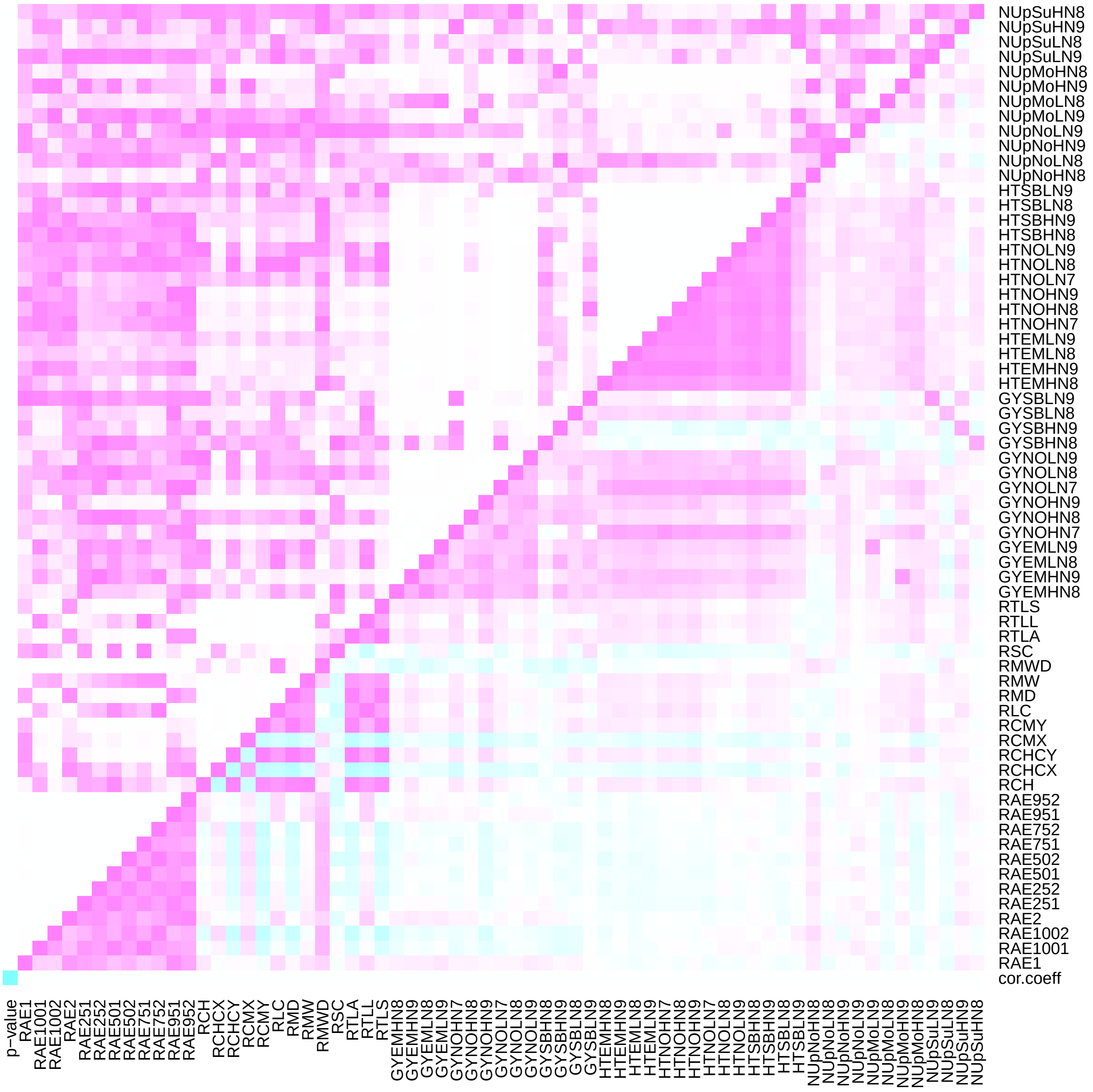

Supplementary Figure 1. Frequency distributions of measured root traits

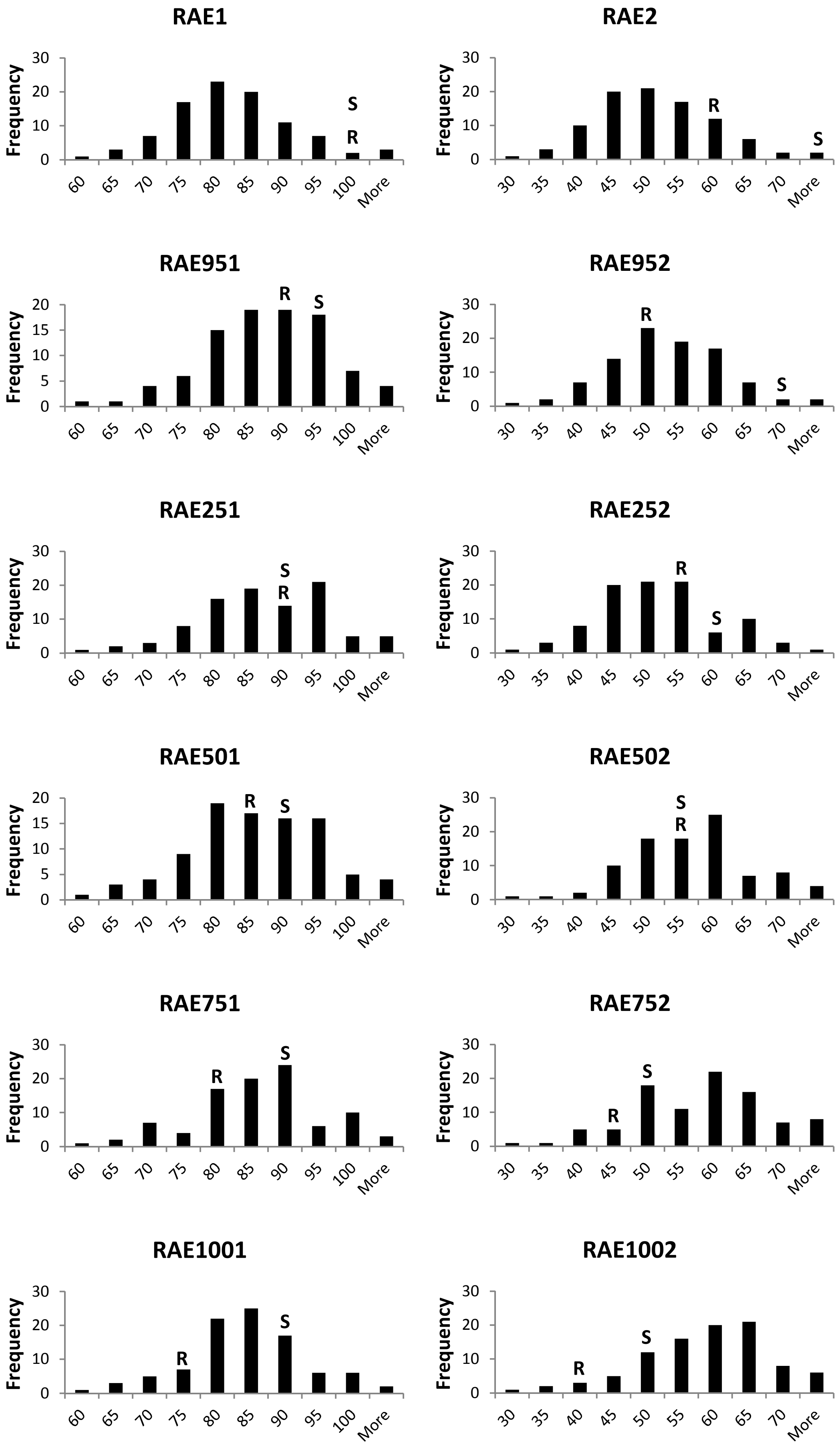

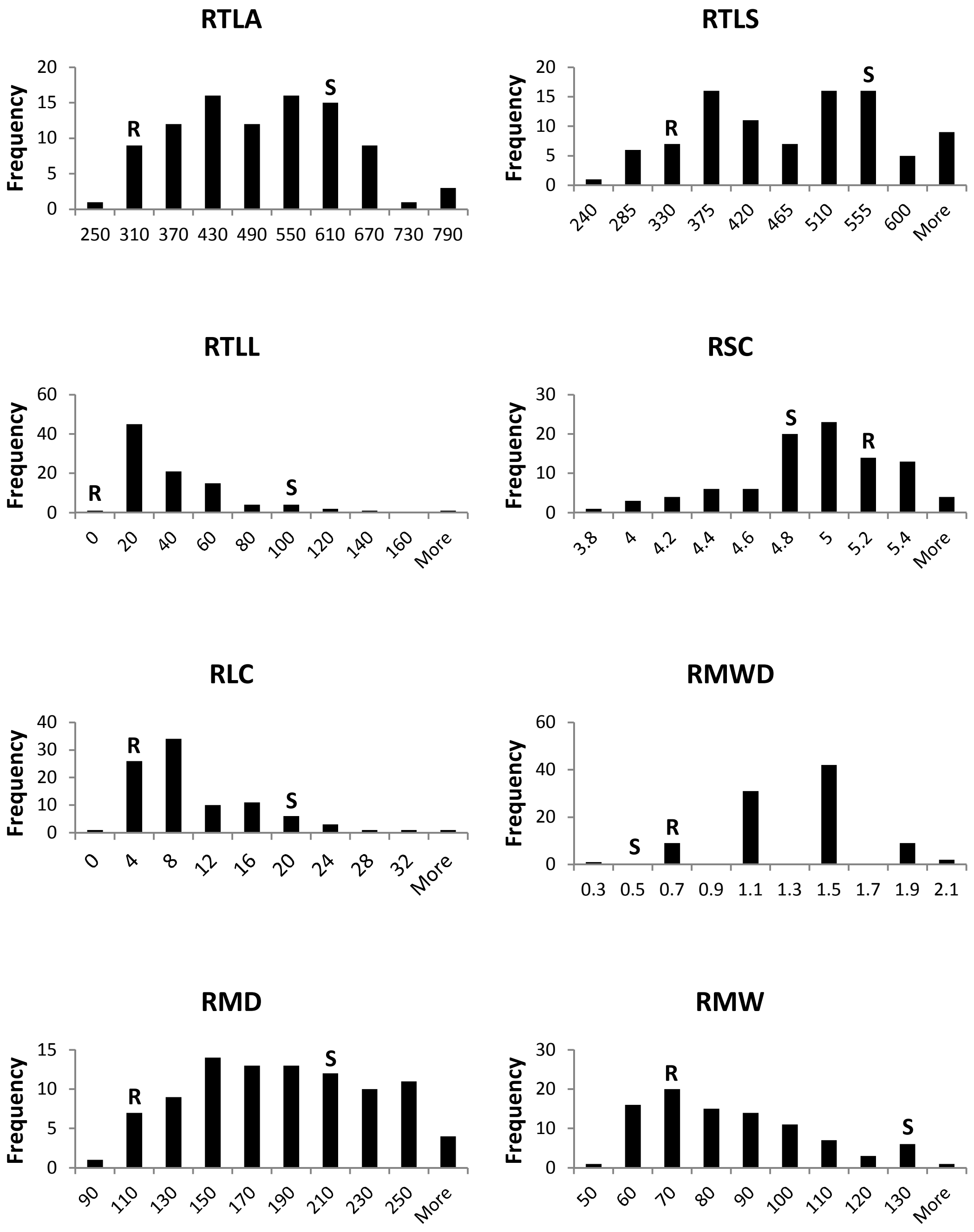
RTLA
Frequency
20
15
10
5
0
250 310 370 430 490 550 610 670 730 790
R
S
RTLS
Frequency
20
15
10
5
0
240 285 330 375 420 465 510 555 600 More
R
S
RTLL
Frequency
60
40
20
0
0 20 40 60 80 100 120 140 160 More
R
S
RSC
Frequency
30
20
10
0
3.8 4 4.2 4.4 4.6 4.8 5 5.2 5.4 More
S
R
RLC
Frequency
40
30
20
10
0
0 4 8 12 16 20 24 28 32 More
R
S
RMWD
Frequency
60
40
20
0
0.3 0.5 0.7 0.9 1.1 1.3 1.5 1.7 1.9 2.1
S
R
RMD
Frequency
15
10
5
0
90 110 130 150 170 190 210 230 250 More
R
S
RMW
Frequency
30
20
10
0
50 60 70 80 90 100 110 120 130 More
R
S

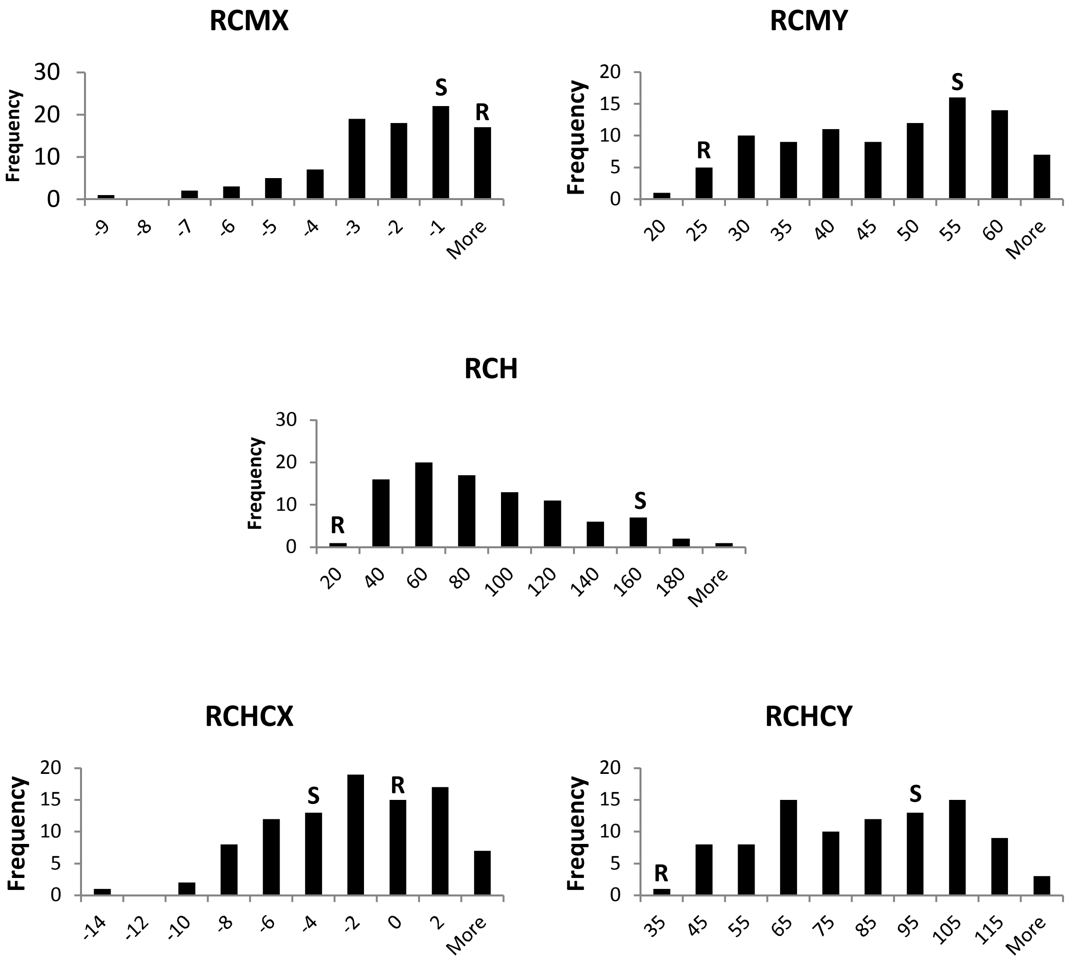
RCMX
Frequency
30
20
10
0
S
R
-9 -8 -7 -6 -5 -4 -3 -2 -1 More
RCMY
Frequency
20
15
10
5
0
R
S
20 25 30 35 40 45 50 55 60 More
RCH
Frequency
30
20
10
0
R
S
20 40 60 80 100 120 140 160 180 More
RCHCX
Frequency
20
15
10
5
0
S
R
-14 -12 -10 -8 -6 -4 -2 0 2 More
RCHCY
Frequency
20
15
10
5
0
R
S
35 45 55 65 75 85 95 105 115 More

Supplementary Figure 2. Distribution of field traits

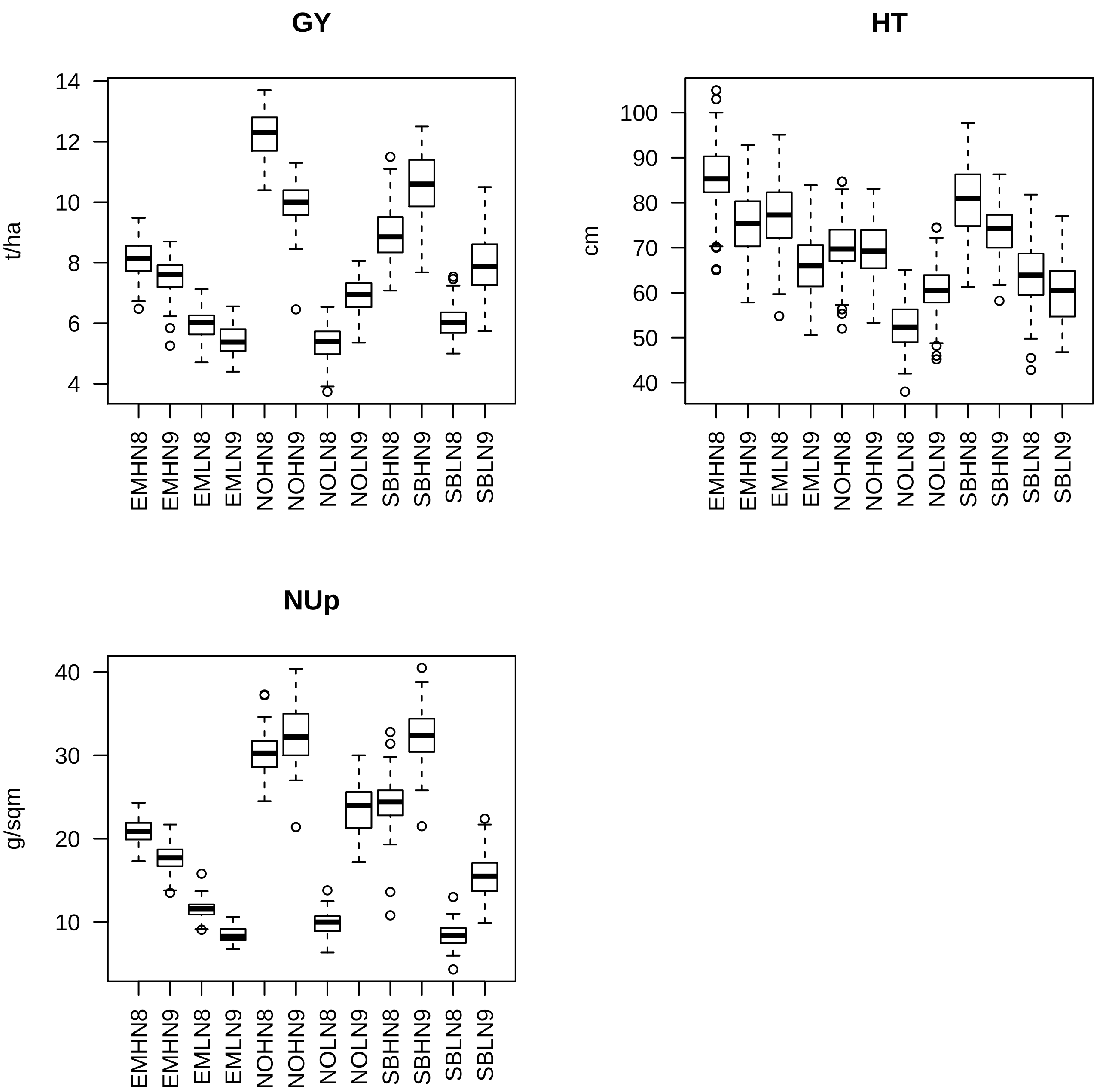

Supplement: Supplementary Data [file supp_erv006_jexbot135848_file001.pdf]
